# Identification, Isolation and Characterization of an Unknown Impurity of Varenicline

**DOI:** 10.3797/scipharm.1201-08

**Published:** 2012-03-20

**Authors:** Balasubramanian Satheesh, Kondayampettai K. Sree Ganesh, Dhandayutham Saravanan

**Affiliations:** 1 Analytical Research and Development, Integrated Product Development, Dr. Reddy’s Laboratories Ltd., Bachupally, Hyderabad-500 072, India; 2 Department of chemistry, National College, Trichy-620 001, India

**Keywords:** Varenicline, Degradant impurity, Preparative HPLC, Isolation, Characterization

## Abstract

An unknown impurity formed during stability sample analysis by a gradient reversed phase ultra-high pressure liquid chromatography (UHPLC) of varenicline tablets at 0.2% level. A simple isocratic preparative method was developed to isolate the unknown impurity with 20 min run time. This unknown impurity was identified and characterized by using spectroscopic techniques. Based on the spectral data, the unknown impurity has been characterized as 4,6,7,8,9,10-hexahydro-1*H*-6,10-methanopyrazino[2,3-*h*][3]benzazepine-2,3-dione. The structure of this impurity was also established unambiguously, prepared by isolation and co-injected into UHPLC to confirm the retention time. To the best of our knowledge, this impurity has not been reported elsewhere.

## Introduction

Varenicline is a highly selective partial agonist of the nicotinic acetylcholine receptor α4β2 subtype [1]. In animal models, the α4β2 nicotinic receptor has been shown to be responsible for the reinforcing properties of nicotine. Both nicotine and varenicline bind to this receptor subtype. Binding of nicotine to this receptor subtype causes dopamine release in the mesolimbic “reward” system (nucleus, accumbens). It is hypothesized that varenicline, a partial agonist, blocks the full-agonist activity of nicotine by competitive binding. As varenicline has this partial agonistic action, it may cause relief of withdrawal and craving symptoms. Withdrawal and craving symptoms are thought to maintain nicotine addiction, and diminishing these symptoms would promote smoking cessation. High performance liquid chromatography (HPLC) is a proven technique that has been used worldwide for many years in controlling the quality and consistency of active pharmaceutical ingredients (APIs) and dosage forms.

Reports on process related impurities [2, 3] and degradation impurities [4] for Varenicline tablets are available in the literature [5]. Additionally, reports on LCMS methods for varenicline are available in the literature [6]. A validated UHPLC method was used [7] for the identification.

It is a mandatory requirement from regulatory authorities to identify and characterize any unknown impurity formed during stability condition which is more than 0.2% in the drug product [8–12]. A comprehensive study has been undertaken to isolate and characterize the unknown impurity by spectroscopic techniques [13–15]. This research paper describes the preparative separation, isolation, identification and characterization of an unknown impurity of Varenicline tablets.

## Experimental

### Chemicals and reagents

The investigated samples of varenicline drug product were obtained from in-house of Dr. Reddy’s laboratories. Methanol, Acetonitrile, acetic acid was of HPLC grade and Ammonium acetate was of Analytical grade procured from Merck (Germany). Water was purified by a Milli-Q-water purification system (Millipore, Bedford, MA, USA) and used for preparation of all the solutions. Pharmaceutical excipients (Microcrystalline cellulose, anhydrous calcium hydrogen phosphate, croscarmellose sodium, anhydrous colloidal silica, magnesium stearate, Titanium dioxide and Iron oxide) were procured from vendor. Dimethyl sulphoxide-*d_6_* (for NMR) was purchased from Aldrich Chemical Co., USA). All solutions were filtered through 0.45 μm membrane filters (Whatman) and degassed by sonication prior to use.

### Ultra performance liquid chromatography (analytical)

The UHPLC system was utilized using a Waters Acquity system equipped with binary solvent delivery pump, an auto sampler and PDA UV detector. The chromatographic separation was performed using a Waters Acquity HSS T3 (150 × 2.1 mm, 1.8 μm), the mobile phase A consists of a mixture of Ammonium acetate buffer (pH 6.5; 0.01 M) and B is a mixture of Ammonium acetate buffer (pH 6.5; 0.01 M), methanol and acetonitrile in the ratio of 10:25:65 (v/v/v). The gradient programme “T (min)/ % B”: 0/2, 1/2, 9/90, 10/90, 10.1/2 and 12/2, with flow rate of 0.35 mL/min was employed. The injection volume was maintained at 5μL and detector set at 235 nm. The column temperature was kept at 40°C.

### Forced degradation of varenicline tablets

Forced degradation studies were performed on the varenicline tablets with the intention of determining the conditions responsible for the formation of the degradation products. Accordingly, degradation studies were conducted by stressing with acid, base, aqueous, peroxide and heat.

#### 

##### Acid stressed degradation

Two hundred milligrams equivalent of varenicline tablets were dissolved in 20 mL of methanol and water in the ratio of 1:1, 5 mL of 0.1N HCl solution was added and refluxed for about 4 h and neutralized.

##### Base stressed degradation

Two hundred milligrams equivalent of varenicline tablets were dissolved in 20 mL of methanol and water in the ratio of 1:1, 5 mL of 0.1N NaOH solution was added and refluxed for about 4 h and neutralized.

##### Aqueous stressed degradation

Two hundred milligrams equivalent of varenicline tablets were dissolved in 20 mL of methanol and water in the ratio of 1:1, the solution was refluxed for about 4 h.

##### Peroxide stressed degradation

Two hundred milligrams equivalent of varenicline tablets were dissolved in 20 mL of methanol and water in the ratio of 1:1, 5 mL of 6% hydrogen peroxide solution was added and maintained at 60°C for 4 h.

#### Thermal stressed degradation

Two hundred milligrams equivalent of varenicline tablets were taken in a Petri dish. Water was sprinkled on the tablets and subjected to 60°C for 3 h.

#### Results of forced degradation study

The degradation samples were analyzed by UHPLC method [7]. Under peroxide stressed conditions, DP-I was formed up to 24%. Attempts were then made to isolate the unknown impurity from peroxide stressed sample.

### High performance liquid chromatography (preparative)

Impurity was isolated from the sample using Shimadzu preparative HPLC Binary system which was equipped with Dual wavelength detector. The data was collected and processed using Millennium software. Approximately 50 mg/mL of sample was prepared to load on to the column. An Inertsil ODS (250 × 20 mm, 10μm) column was employed for the separation of DP-I. The mobile phase is consisting of a mixture of 0.02M ammonium acetate buffer, with the pH adjusted to 6.5 with ammonia and methanol in the ratio of (70:30). The flow rate was kept at 15 mL/min. Detection was carried out at 235nm.

### Sample preparation

The varenicline sample was prepared at a concentration of 1 mg/mL in methanol for the analytical UHPLC and 50 mg/mL for the preparative HPLC analysis.

### UHPLC-ToF MS

The UHPLC-ToF MS system consisted of an ACQUITYTM Ultra Performance Liquid Chromatography system and a Micro mass LCT Premier XE Mass Spectrometer (High sensitivity orthogonal time-of-flight instrument, Waters, Modiford, USA) equipped with a lock mass sprayer, operating in either the positive or negative ion mode. All analyses were acquired using the lock spray to ensure accuracy and reproducibility; leucine-enkephalin was used as the lock mass. Sample of concentration 0.02mg/mL in methanol was infused in ToF MS at a flow rate of 10μL/min. High resolution (W mode, FWHM 10500) positive polarity scan responses were collected from m/z 100 to 1000 at a rate of 1.0s/scan.

### NMR spectroscopy

The NMR experiments were performed on Varian spectrometer operating at 400 MHz, Mercury plus, in DMSO-*d*_6_ & (DMSO+D_2_O for DP-I) at 25°C. The proton and carbon chemical shifts were reported on δ scale in ppm, relative to TMS (δ=0.00ppm) and DMSO (δ=39.50ppm) as internal standard, respectively.

### FT-IR spectroscopy

IR spectra were recorded in solid state as KBr dispersion medium using Perkin-Elmer FT-IR spectrophotometer.

## Results and Discussion

### Detection of DP-I

**Fig. 1.**
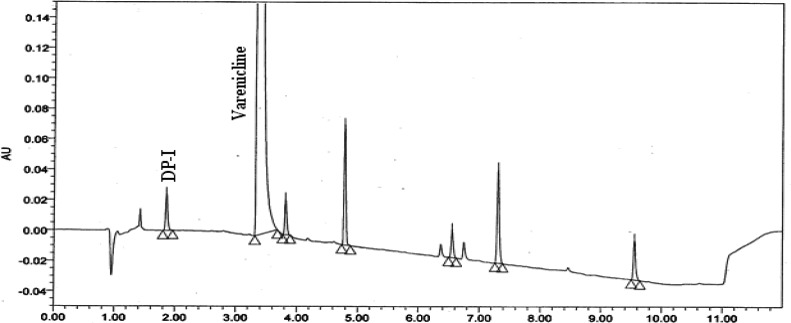
UHPLC stability chromatogram of Varenicline tablet sample.

**Fig. 2.**
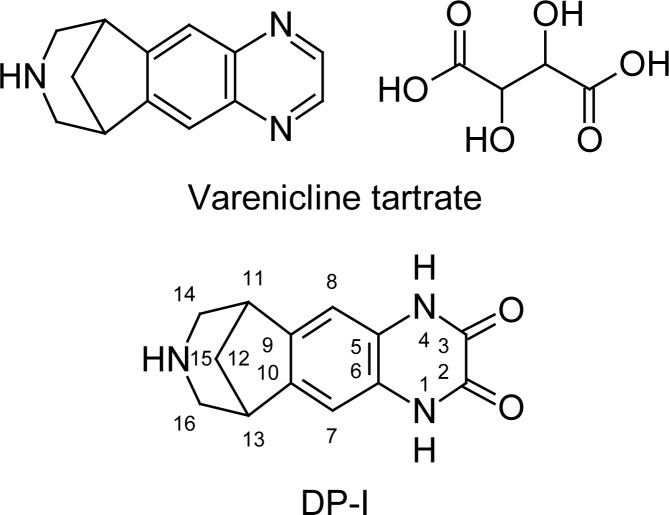
Chemical structures of Varenicline Tartrate and degradant product (DP-I).

**Fig. 3.**
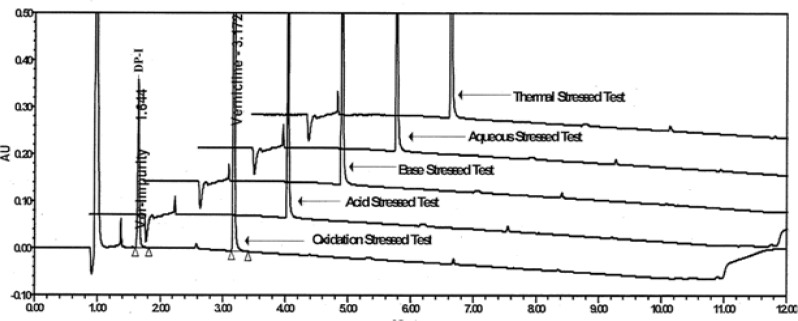
Comparison of chromatograms obtained from the forced degradation studies.

A typical analytical UHPLC chromatogram of a stability sample of Varenicline drug product recorded using the UHPLC method [7] as described are shown in Fig. 1. The target impurity under study is marked as DP-I retention time (RT): 1.857 & Varenicline (retention time (RT): 3.354. The structure of the DP-I and varenicline are shown in Fig. 2. The representative UHPLC chromatograms of the forced degradation study are shown in Fig. 3. The DP-I is polar with respect to varenicline.

### Isolation of impurity by preparative HPLC

Several trials were performed to achieve the required resolution. Finally an isocratic solvent system was developed with good resolution and short runtime. Desaltification procedure was adopted to isolate the impurity as pure fractions. The impurity fractions were collected from several injections and then pooled. These fractions were concentrated separately by using Rotavapor (Heidolph Laboratory 4002 control) under high vacuum. The aqueous solutions were subjected to lyophilization to solidify the impurity.

### Characterization of varenicline DP-I

**Fig. 4.**
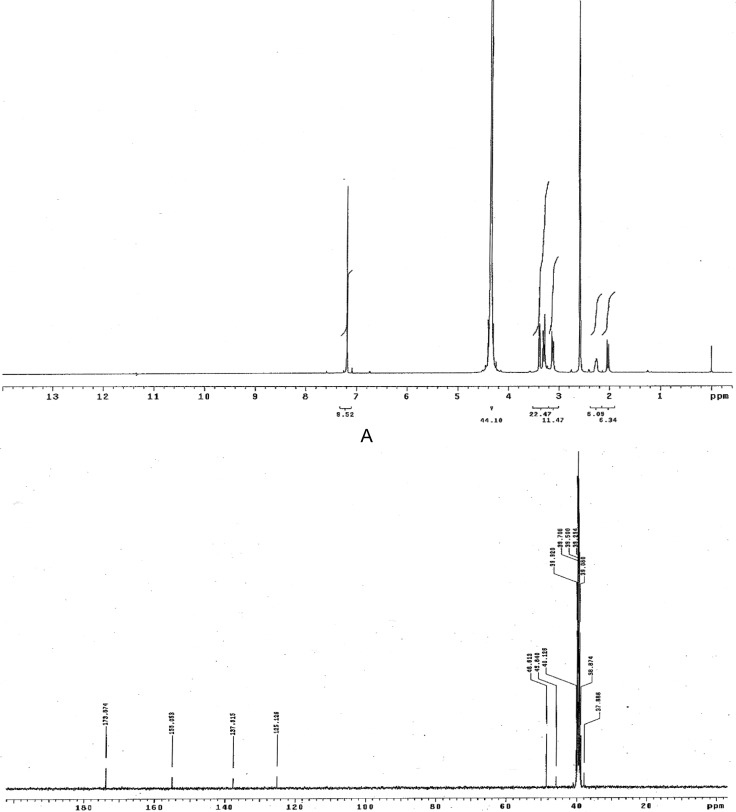
(A) ^1^H NMR spectrum of DP-I. (B) Proton decoupled ^13^C NMR spectrum of DP-I.

**Fig. 5.**
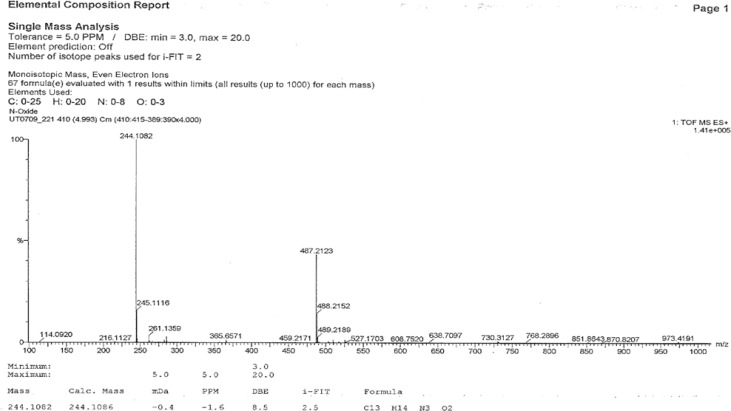
UHPLC-ToF MS^+^ of DP-I.

The ^1^H NMR and ^13^C NMR spectra of DP-I are shown in Fig. 4 (A) & (B), respectively. The NMR and High resolution mass spectroscopic (HR-MS) data of the isolated impurity was compared with those of Varnecline tartrate data (C_13_H_13_N_3_). The proton NMR of isolated impurity was similar to that of parent compound. The mass (ToF-MS), FT-IR and ^13^C NMR spectrum reveals important structural insight of the DP-I. The positive HR-MS data Fig. 5 of impurity DP-I has exhibited protonated molecular ion [M+H]^+^ at 244, which corresponds to the molecular formula C_13_H_14_N_3_O_2_. The molecular formula shows that the impurity has two oxygen atoms more than the parent compound. The IR spectrum shows the stretching vibration at 1696 cm^−1^ reveals the presence of amide carbonyl (-CONH) and the ^13^C NMR also confirms the amide carbonyl at 173.8 ppm. The above spectral data confirms the oxidation of pyrazine ring.

The impurity obtained as pale white crystals. mp 71–73. RP-UHPLC, tR = 1.8 min (98.5% purity). MS (ESI, 70 eV): [M + H^+^] m/z 244. FT-IR (KBr), v, cm^−1^ 3371, 3319, 3279, 3173, 3005, 2808, 1696, 1678, 1588, 1406, 1388, 1338, 1305, 1264, 1135, 1067, 873, 790, 680, 569, 485. ^1^H NMR (400 MHz, DMSO-*d_6_* *+* D_2_O, TMS): δ 7.2 (s, 2H, H-7,8), 3.1–3.4 (m, 6H, H-11,13,14 & 16),2.3 (m, 1H, H-12), 2.0 (d, 1H, 11.2 Hz, H-12).^13^C NMR (100 MHz, DMSO-*d_6_*, TMS): δ 173.8 (C-2,3), 155.1 (C-5,6), 137.6 (C-9,10), 125.1 (C-7,8), 38.8 (C-11), 37.9 (C-12), 38.8 (C-13), 45.8 (C-14), 48.6 (C-16). UHPLC ToF MS+: m/z [M + H^+^].Calcd for C_13_H_13_N_3_O_2_: 244.1086; found: 244.1082.

Based on the above spectral data, the molecular formula of DP-I is C_13_H_13_N_3_O_2_ and the corresponding structure was characterized as 4,6,7,8,9,10-hexahydro-1*H*-6,10-methanopyrazino[2,3-*h*][3]benzazepine-2,3-dione.

## References

[1] www.emea.europa.eu/humandocs/PDFs/EPAR/champix/H-699-en6.pdf

[2] Busch FR, Concannon PE, Handfield RE, McKinley JD, McMahon ME, Singer RA, Watson TJ, Withbroe GJ, Stivanello M, Leoni L, Bezze C (2008). Synthesis of (1 (Aminomethyl)-2,3-dihydro-1H-inden-3-yl)methanol: Structural Confirmation of the Main Band Impurity Found in Varenicline® Starting Material. Synth Commun.

[3] Varenicline standards and impurity controls. www.freepatentsonline.com/US2007/0224690.html

[4] N-formyl and N-methyl degradation products. www.freepatentsonline.com/y2004/0235850.html

[5] Methods of reducing degradant formation in pharmaceutical compositions of Varenicline. www.freepatentsonline.com/y2008/0026059.html

[6] Varenicline standards and impurity controls. www.freepatentsonline.com/EP2004186.html

[7] Satheesh B, Kumarpulluru S, Raghavan V, Saravanan D (2010). UHPLC Separation and Quantification of Related Substances of Varenicline Tartrate Tablet. Acta Chromatogr.

[8] ICH harmonized tripartite guideline. Q3A (R2). Current step 4 version dated 25 October 2006

[9] ICH harmonized tripartite guideline. Impurities in New Drug products Q3B (R2). Current step 4 version dated 2 June 2006.

[10] ICH harmonized tripartite guideline. Stability testing of new drug substances and Products Q1A (R2). Current step 4 version dated 6 February 2003.

[11] US Food and Drug Administration. Drug Stability Guidelines, February 1987.

[12] United States Pharmacopeia. 25, US Pharmacopeial Convention, Rockville, MD 2000, 7, General Notices.

[13] Ahuja S, Alsante KM (2003). Handbook of isolation and characterization of impurities in pharmaceuticals.

[14] Smith RJ, Webb ML (2007). Analysis of Drug Impurities.

[15] Ahuja S Impurities Evaluation of Pharmaceuticals.

